# A Systematic Review and Meta-Analysis of Associations between Green and Blue Spaces and Birth Outcomes

**DOI:** 10.3390/ijerph17082949

**Published:** 2020-04-24

**Authors:** Selin Akaraci, Xiaoqi Feng, Thomas Suesse, Bin Jalaludin, Thomas Astell-Burt

**Affiliations:** 1 Population Wellbeing and Environment Research Lab (PowerLab), School of Health and Society, Faculty of Social Sciences, University of Wollongong, Wollongong, NSW 2522, Australia; sa066@uowmail.edu.au (S.A.); xiaoqi.feng@unsw.edu.au (X.F.); 2 School of Public Health and Community Medicine, Faculty of Medicine, University of New South Wales, Sydney, NSW 2052, Australia; 3 Menzies Centre for Health Policy, University of Sydney, Sydney, NSW 2006, Australia; 4 National Institute of Environmental Health, Chinese Center for Disease Control and Prevention (China CDC), Beijing 102206, China; 5 NIASRA, National Institute for Applied Statistics Research Australia School of Mathematics and Applied Statistics, Faculty of Engineering and Information Sciences, University of Wollongong, Wollongong, NSW 2522, Australia; tsuesse@uow.edu.au; 6 Ingham Institute for Applied Medical Research, University of New South Wales, Sydney, NSW 2170, Australia; b.jalaludin@unsw.edu.au; 7 School of Public Health, Peking Union Medical College and The Chinese Academy of Medical Sciences, Beijing 100730, China

**Keywords:** green space, blue space, pregnancy outcomes, health benefits, urban planning

## Abstract

Previous studies suggest that green and blue spaces may promote several health outcomes including birth outcomes. However, no synthesis of previous work has specifically asked policy-relevant questions of how much and what type is needed in every neighborhood to elicit these benefits at the population level. A systematic review and meta-analyses were conducted to synthesize thirty-seven studies on the association between residential green and blue spaces and pregnancy outcomes. Meta-analyses were performed for birth weight (BW), small for gestational age (SGA), low birth weight (LBW) and preterm birth (PTB). Increase in residential greenness was statistically significantly associated with higher BW [β = 0.001, 95%CI: (<0.001, 0.002)] and lower odds of SGA [OR = 0.95, 95%CI: (0.92, 0.97)]. Associations between green space and LBW and PTB were as hypothesized but not statistically significant. Associations between blue spaces and pregnancy outcomes were not evident. No study explicitly examined questions of threshold, though some evidence of nonlinearity indicated that moderate amounts of green space may support more favorable pregnancy outcomes. Policy-relevant green and blue space exposures involving theory-driven thresholds warrant testing to ensure future investments in urban greening promote healthier pregnancy outcomes.

## 1. Introduction

The plausible benefits of residential green [1,2,3,4,5,6,7,8,9,10] and blue spaces [11,12,13,14] on health, including perinatal health, have gained increasing attention over the last 10 years. General reviews of prior work on green space and health have already been published [15,16,17,18]. Three reviews on green space and pregnancy outcomes have also been published [19,20,21]. The study carried out by Dzhambov et al. (2014) performed a meta-analysis of eight studies of association between residential greenness and birth weight. Subsequent work by Banay et al. (2017) qualitatively synthesized the findings of 14 studies of association between residential greenness and pregnancy outcomes. The most recent review [21] included a meta-analysis of 36 studies. Each review concluded that there is increasing evidence of an association between green space and healthier pregnancy outcomes. However, none of these reviews examined the studies of blue space and birth outcomes. This is an important gap, as aquatic environments are often important dimensions including within green space quality assessments (e.g., [22,23]), due to their influences on landscape preferences and human health [24,25]. The abovementioned reviews also did not emphasize the distinct and policy-relevant influences of different exposure metrics on pregnancy outcomes.

For research on green and blue space and pregnancy outcomes to be more consequential, it must be translated and integrated within planning guidelines, and monitoring of their implementation should be conducted thereafter. This depends, to some extent, upon identifying critical thresholds of exposure at which healthier pregnancy outcomes are more likely to occur. Our objective was to systematically review and meta-analyze prior research, paying close attention to the exposure methods and measures, to identify the critical thresholds of green and/or blue space supportive of healthy pregnancy outcomes.

## 2. Domain Pathways Linking Green and Blue Space with Healthier Pregnancy Outcomes

Green spaces are hypothesized to influence health through three potential pathways: (1) mitigation; (2) restoration; (3) instoration [26]. The benefits of green spaces on pregnancy outcomes plausibly operate through the same mechanisms [27] (Supplementary Document 1, Figure S1). Intuitively, pregnancy shifts how women interact with green and blue spaces in ways that could turn up or turn down the flow of benefits along particular domain pathways. This consideration led us to the following two points that warrant brief attention. 

First, pregnancy may modify preferential choices and perceived restorative potential with regards to the size and quality of discrete parks. For example, in terms of green space, previous studies have suggested that larger parks within a short walking distance of home (e.g., 1 km) are more attractive for walking and more vigorous physical recreation [28,29,30]. But these findings may not generalize well for pregnant women, for whom the presence of a green space that they perceive as safe, containing good seating, restorative features (e.g., blue space) and an easy to walk for purposes of relaxation and meeting friends, may be preferred over a sports oval. In other words, the perceived qualities of parks may become more salient during pregnancy, determining the frequency of use and modifying restorative potential.

Secondly, the mitigation domain pathway may take on additional salience during pregnancy, due to the widely reported links between air pollution and unfavorable pregnancy outcomes. Although this affects all people within areas containing heavy traffic to some degree, evidence suggests that the mother and baby may be more susceptible, due to high cell proliferation [31,32], immature organ system and mechanism for detoxification [31,32] of the fetus, as well as increased tidal volume in pregnant women [32]. Green and blue spaces—perhaps especially within the context of coastal communities—may play an important role in reducing harms due to poor air quality.

From these considerations of the three domain pathways and how they may apply in subtly different ways during pregnancy and for related health outcomes, we made the following preliminary conclusions that were then used in benchmarking papers covered in this review:

(i) Associations between pregnancy outcomes and green space quantity may be strongly contingent upon the quality of those spaces as perceived by pregnant women, so indicators of quantity and quality warrant explicit investigation simultaneously within the context of the restoration and instoration domains;
(ii) The importance of the mitigation domain pathway, and of air pollution especially, may be amplified, due to increased biological susceptibility among women living within contexts where there is heavy traffic.

## 3. Method

### 3.1. Selection Protocol

The review was conducted in accordance with the PRISMA guidelines and covered peer-reviewed journal articles on the effects of green and blue spaces on pregnancy outcomes. Pregnancy outcomes included both infant and maternal health outcomes during perinatal and neonatal stages. We used the following free-text term keyword combinations: “green space” and “blue space” and “pregnancy outcomes” (Supplementary Document 1, Table S1). 

Two reviewers (SA and JC) conducted the search protocol independently with predefined search terms and strategies from databases of the published peer-reviewed literature—Science Direct, PubMed, ProQuest, Scopus and Google Scholar—in November and December 2018. Additionally, the snowballing technique, scanning reference lists of relevant publications, was used to retrieve as many related publications as possible. Peer-reviewed journal articles, written in English, were included, regardless of time limitation. Animal studies and commentary/review studies were excluded.

### 3.2. Quality Assessment

The quality assessment tool for observational cohort and cross-sectional studies by the National Heart, Lung, and Blood Institute (NIH) was used to evaluate the qualities of all included articles [33]. The tool includes 14 criteria referring to several aspects of studies, including the efficiency of the sample population, objectiveness of exposure and outcome variables. For each item in the list, three options for answers are suggested which are “Yes”, “No” or “Other” (CD, NR, NA). If the criteria was met (Yes) it is assigned to value of 1, whereas if the criterion was not met (No), was not clear (CD), was not reported (NR) or was not applicable (NA) 0 points were assigned. Finally, the overall score was calculated by dividing the sum of the positive scores by the total number of applicable questions. The scores below 50, between 50 and 74 or above 75 meant the articles were regarded as low, fair and high quality, respectively. The same classification was also used previously [19]. Two reviewers (SA and JC) conducted the quality assessment independently. Results and disagreements were discussed, and discrepancies were resolved. 

### 3.3. Meta-Analysis

Meta-analysis was limited to those perinatal health outcomes for which at least 5 studies were available. Four separate meta-analyses were conducted for the association between green space exposure and four perinatal outcomes: birth weight (BW), preterm birth (PTB), small for gestational age (SGA) and low birth weight (LBW). The quality effects (QE) model [34] in MetaXL version 5.3 [35] was used. We extracted effect size estimates only for adjusted models that included the general study population, disregarding any stratified subgroups. 

Four different approaches were performed in which exposure was treated differently. In the first approach, all studies were included, disregarding the differences in types of exposure measures and normalization of amount of increase. In the second approach, we included only those studies that measured the Normalized Difference Vegetation Index (NDVI) (n = 18), which indicates the density of greenness in a certain area, which is sourced from a satellite image [36]. In the third approach, we calculated the effect estimates for an increment of 10% all green space measures (Supplementary Document 1, Tables S6, S11, S16 and S21). Finally, we reported the effect estimates based on 0.1 unit increase of NDVI (Supplementary Document 1, Tables S8, S13, S18 and S23). We reported effect estimates corresponding to increase in NDVI if the study measured greenness in multiple ways in order to obtain comparable results. If the study did not measure NDVI, but tested a different greenness variable, we considered the effect size corresponding to the alternative measure. We examined the effect sizes corresponding to 300 m or the closest buffer size reported, since it captures residential greenness, and also it allowed comparability between studies. A study [37] that measured green space percentage in census unit and did not define buffer distances was also included. Effect estimates based on continuous NDVI were included when other types of exposure (e.g., Q4 vs. Q1) were also reported [10,38]. If a study calculated effect sizes separately for urban and suburban areas, we considered the one reported for urban areas.

The main analysis for BW was based on standardized regression coefficients (β). We combined BW that are adjusted for gestational age and term birth weight (TBW) for the analysis, as well as conducting a separate meta-analysis for TBW (Supplementary Document 1, Table S25) and BW (Supplementary Document 1, Table S26) that is adjusted for gestational age. For Cusack et al. (2017), we reported effect sizes corresponding to two cities as different units of analysis. Similarly, for Dzhambov et al. (2019), effect sizes corresponding to UIT (included 1280 children and conducted in Lower Inn Valley, Austria in 1998) and BBT (included 1251 children and conducted in Wipp Valley around the Brenner Pass, Austria and Italy in 2004/2005) surveys were treated as separate studies in the meta-analysis. For Richardson et al. (2017), we considered effect size estimates for the between-mothers’ model. For Laurent et al. (2013), we included a regression coefficient corresponding to the model adjusted for NOx estimated via the CALINE4 dispersion model (150 m). 

The main analysis for LBW was based on odds ratios. The study by Dadvand et al. (2014a) was excluded, since green space was not the main exposure in this study. The study by Seabrook et al. (2019) was also not included, since the buffer distance used in the study (1600 m) was not consistent with other studies. We combined LBWs that are adjusted for gestational age and term low birth weight (TLBW) for the analysis, as well as conducted separate analysis for TLBW (Supplementary Document 1, Table S27) and LBW (Supplementary Document 1, Table S28). When the LBW is measured as a dichotomous outcome such as LBW (<2500 g) or very low birth weight (VLBW) (<1500 g) [39,40], we extracted the effect estimate of LBW. 

The main analysis for SGA was based on odds ratios. As for LBW, the study by Dadvand et al. (2014a) was excluded, since the greenspace was not the main exposure in this study. For the study by Casey et al. (2016), we extracted the effect size estimate that was reported for cities. 

The main analysis for preterm birth was based on odds ratios. The study of Donovan et al. (2011) was not included, since the result for PTB was not shown in the study. Likewise, in the case of LBW, we extracted the effect size estimates reported for moderate PTB when it was also reported for very preterm birth (VPTB) to increase comparability between studies. For the study of Glazer et al. (2018), we reported the effect size estimate that was associated with adjustment for markers of neighbourhood socioeconomic status (SES).

Heterogeneity among studies were assessed by chi-square tests. I2 statistics were used to quantify the heterogeneity. According to the thresholds of I2, heterogeneity is considered as small (0% to 40%), moderate (30% to 60%), substantial (50% to 90%) and considerable (75% to 100%) (https://handbook-5-ochrane.org/chapter_9/9_5_2_identifying_and_measuring_heterogeneity.htm).

## 4. Results

The initial searches generated 1879 results. Six additional articles were identified through a Google Scholar search and reference lists of the articles. After removing duplicates, 1534 articles remained for the title and abstract scan. Full text of 94 articles were reviewed. After including the relevant articles, which were published after concluding the initial search, 37 studies were selected for this systematic review and meta-analysis (Supplementary Document 1, Figure S2). 

### 4.1. Study Characteristics

Articles were published over the last decade and were conducted in high-income Western countries, except for the four studies from Israel [39,40] (Supplementary Document 1, Table S3) and China [41,42]. Most of the studies (n = 30) utilised a cross-sectional study design, while remaining studies were cohort [38,41,43,44], case-control [42], ecological [27,45] or quasi-experimental in design [46]. The sample size varied from 427 (min) to 3,026,603 (max). The studies obtained outcome data through official birth records. The sample size was representative in most of the studies. However, some studies did not report this information and two studies [47,48] indicated that the sample size might not be representative. All articles fell into the category of medium-high quality (see Supplementary Table S2).

#### 4.1.1. Exposure Variables

The most commonly used (n = 27) green space measure was NDVI within multiple circular buffer distances (50–2000 m) around the residential address of each mothers or centroid of each area of residence (Table S4). Most of the studies (n = 26) evaluated mean value of NDVI, while one study [39] also considered variance of greenness. Some studies considered the distance to major green spaces in the neighbourhood, either by calculating the linear distance [38,49,50,51,52,53] or by using street network buffer based on walkable distances [40,49,50,54]. Some studies [27,37,44,45,46,55,56,57,58,59] used the proportion of green/natural spaces in the total area of census block or zip code as a surrogate for greenness exposure. A number of studies [27,45,51,56] considered public green spaces, while one [46] also included private greenness (e.g., private gardens) in the analysis. 

Measures of specific types of vegetation such as the percentage of tree coverage [39,54,60,61,62] and street tree buffering (availability of trees on the street) [61,63] were also assessed in some studies. Finally, some studies [46,52,53,64,65] assessed the association between blue space and pregnancy outcomes, either by using residential proximity to fresh water bodies [52,53,64,65] and coasts [52,53], or analysing the proportion of water surfaces within residential areas [46]. A recent study from the United States [54] used a composite measure, urban neighbourhood green index (NGHI), which comprised green space percentage and proximity measures, as well as building density and height of surrounding structures, hypothesizing these to be structural influences on green space quality. While most studies measured exposure only at the time of birth, some [66,67] considered seasonal changes and the critical periods of pregnancy by measuring greenspace multiple times [39,43,60,68].

#### 4.1.2. Outcome Variables

All studies used objective and validated outcome variables. A major number of studies (n = 32) investigated infant related birth outcomes while fewer studies [37,41,45,51,53,69,70] focused on maternal health outcomes, including pre-eclampsia [45,53,70], hypertension [53], gestational diabetes [41,45,53] and depression [37,51]. The number of studies that examined foetal growth (BW, LBW, and SGA), gestation length (gestational age, PTB, VPTB) and other outcomes (blood pressure, head circumferences, 5 min low Apgar score, neonatal mortality) were 28, 16 and 7, respectively (Supplementary Document 1, Table S4).

#### 4.1.3. Potential Effect Modifiers

Individual characteristics (e.g., age of the mother, sex of the infant) were considered in most of the studies. Nevertheless, only a few studies were able to control for components of the built environment. For example, the population density in the residential area and air pollution were taken into account by seven studies [10,46,47,57,61,67,70], while temperature [47] and noise [10,47,57,69] received less attention. Many studies [37,38,39,40,47,51,52,56,63,64,67] considered relationship status a plausible marker of social support. Duration of residency, which provides information on potential duration of exposure, was considered by only three studies [38,46,56].

### 4.2. Meta-Analysis

**Figure 1:**
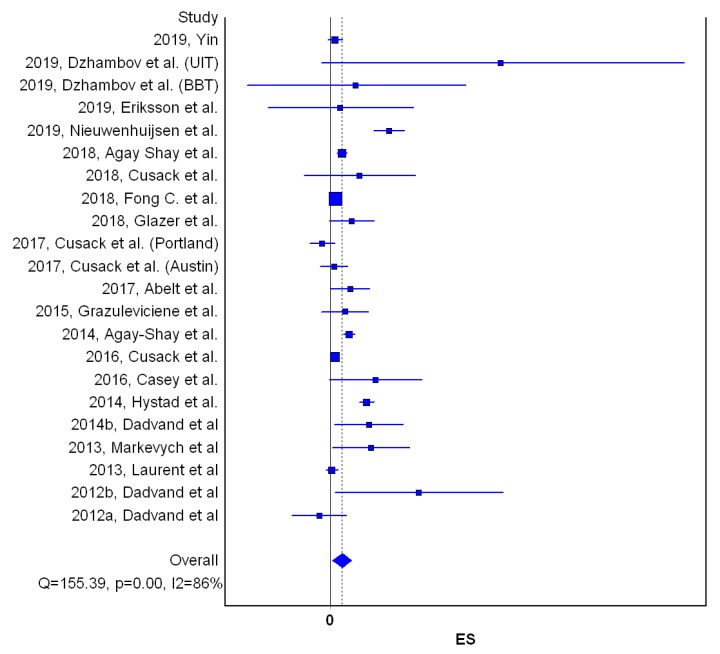
Forest plot on the effect of residential greenness (NDVI) on birthweight (standardized regression coefficients, quality effects model). Note: ES—effect size; Q and I2—heterogeneity statistics. Horizontal lines, square and diamond symbols are indicating confidence intervals, effect estimates, and overall effect, respectively.

Meta-analysis on BW, LBW, SGA and PTB included twenty, fourteen [38,39,40,47,64,65,67], eleven [10,38,39,47,50,52,62,64,66,67,68,71] and eleven studies [10,38,39,40,52,64,66,68,72], respectively. In adjusted models, the increase of residential greenness (NDVI) within 250 m (or 300 m) buffer was weakly but statistically significantly and positively associated with higher BW [β = 0.001, 95%CI: 0.000, 0.002) (Table 1 and Figure 1). 

**Figure 2:**
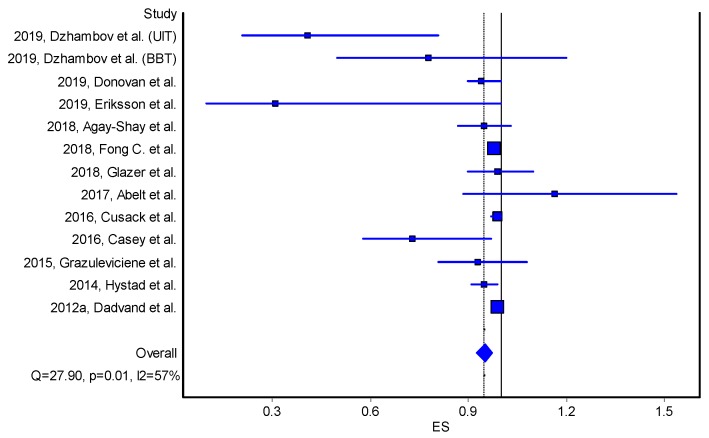
Forest plot on the effect of residential greenness (NDVI) on small for gestational age (odds ratios, quality effects model). Note: ES—effect size; Q and I2—heterogeneity statistics. Horizontal lines, square and diamond symbols are indicating confidence intervals, effect estimates and overall effect, respectively.

Results were similar when other studies, which measured residential greenness via green space percentage [46,47,56], were included (result is not shown). Weak, positive and statistically significant association remained when we calculated the effect estimates corresponding to a 10% increase in residential greenness (NDVI and green space percentage) (Supplementary Document 1, Table S7). However, statistical significance was attenuated when we adjusted the size of effect estimates based on a 0.1 increase in NDVI within 250 m/300 m (Supplementary Document 1, Table S9). Residential greenness within 250 m (or 300 m) buffer was weakly but statistically significantly and positively associated with reduced odds of small for gestational age [OR = 0.95, 95%CI (0.92, 0.97)] (Table 2, Figure 2).

**Figure 3:**
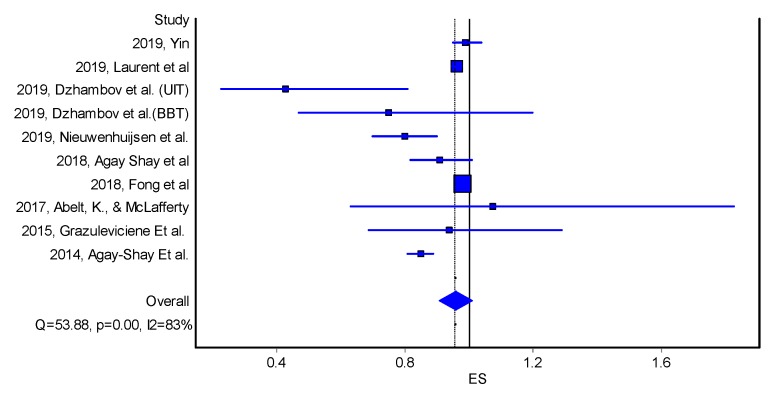
Forest plot on the effect of residential greenness (NDVI) on low birth weight (odds ratio, quality effects model). Note: ES—effect size; Q and I2—heterogeneity statistics. Horizontal lines, square and diamond symbols are indicating confidence intervals, effect estimates and overall effect, respectively.

Reduced odds of LBW was also associated with an increase in residential greenness, however, the association was not statistically significant [OR = 0.96, 95%CI 091, 1.01] (Table 3 and Figure 3). 

**Figure 4:**
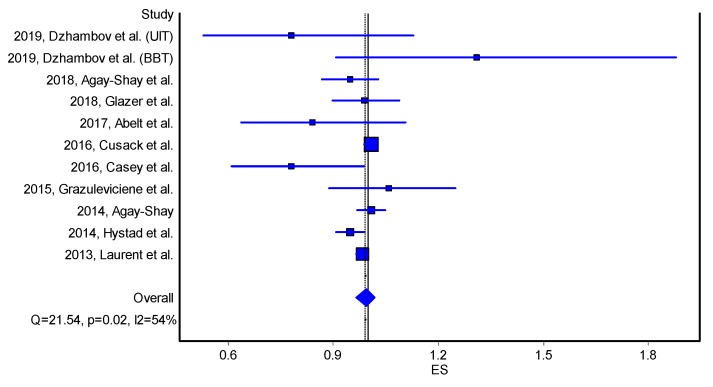
Forest plot on the effect of residential greenness (NDVI) on preterm birth (odds ratio, quality effects model). Note: ES—effect size; Q and I2 —heterogeneity statistics. Horizontal lines, square and diamond symbols are indicating confidence intervals, effect estimates and overall effect, respectively.

Similarly, there was statistically non-significant borderline reduction in the odds of PTB [OR = 0.99, 95%CI 0.97, 1.02] corresponding to an increase in green space (Table 4 and Figure 4). 

We observed similar results when we included only those studies in which NDVI was used as green space proxy and when we calculated effect estimates based on a 10% increase in residential greenness (Supplementary Document 1, Tables S12, S17 and S22) and a 0.1 unit increase in NDVI (Supplementary Document 1, Tables S14, S19 and S24). 

Publication bias was detected through application of the Funnel plots and Doi plot [73] methods. Both methods detected the presence of a publication bias (Supplementary Document 1, Figures S3–S10). 

### 4.3. Non-Pooled Outcomes and Exposures

#### 4.3.1. Associations with Percentage of Green Space

Evidence of an association between feotal growth and the proportion of green space was observed [46,56,61], as well as for green space and gestational length [56] among newborns of mothers with lower educational attainment. An interquartile range increase in the percentage of green space within 5 km from the residential address of each mother was associated with non-significant lower neonatal blood pressure [59].

#### 4.3.2. Associations with Tree Canopy

Six studies looked into the potential effects of living in the vicinity of trees on foetal growth [39,54,61,62,63,64]. Four studies [54,61,63,64] reported a statistically significant association between living in a neighbourhood with more trees and increased BW in unadjusted models. Three studies [54,62,64] observed statistically significant evidence of association in adjusted models. One study [54] found a statistically significant association between tree canopy and BW, but only among high SES groups. Another study reported that the risk of term LBW associated with proximity to major roads was reduced within the context of higher quantity of tree canopy within 200 m [63]. Similarly, a 10% increase in tree canopy within 50 m was associated with decreased risk of SGA by 1.42 per 1000 births [62]. Findings of another study [61], which compared two cities, showed that greater number of trees within 1000 m buffers from mother’s residence was associated with 14.7 g (95% CI: −23.0, −6.3) lower BW from data in Austin, but no statistically significant association from data in Portland [61]. One study [64] reported that an increase in street trees within 100 m, 250 m and 500 m buffers was associated with 0.11%, 0.02% and 0.01% statistically significant decreases in the odds of PTB risk, respectively. However, another study [62] did not show any statistically significant association.

#### 4.3.3. Associations with Distance to Green Space

Seven studies evaluated potential influence of proximity to major green spaces on foetal growth [38,40,49,50,51,52,61], of which three [40,49,52,61] observed statistically significant positive association in unadjusted models; the association remained statistically significant in adjusted models in two studies [40,52]. Living within 300 m around a major green space was found to be associated with a 8.3 g (Cl%95 = 1.1, 15.5) [61] and 22.8 g (95% CI: 1.26, 44.3) [49] statistically significant increase in BW in unadjusted models, but not in adjusted models [40,49]. Living closer to green spaces was also associated with a lower risk of LBW in unadjusted [38,40] and adjusted models [40]. A study [38] reported increase odd of LBW (OR 2.23, 1.20-4.15) and TLBW (OR 2.97, 1.04-8.45) among women who live within a long distance (>1000 m) from the park. Whereas, some studies reported no statistically significant association between proximity to greener areas and LBW [50,64] or small for gestational age [64]. Furthermore, another study reported a statistically significant association between living within 800 m of space and decreased BW [54]. Similarly, a small inverse association between living close to major green space and BW among those with only the secondary school education has been reported [50]. 

Five studies examined the potential influence of proximity to major green space/recreational place on gestation length [38,40,50,52,64]. Only one study [52] reported a statistically significant 7% decrease in the odds of PTB among mothers who lived within 500 m from major green spaces, when the model was adjusted for SES and the demographic characteristics of participants. The remaining studies did not demonstrate a statistically significant association.

#### 4.3.4. Quality of Green Space

A study [54] has reported statistically significant positive association between NGHI measures and birth weight. Another study has shown evidence suggestive that landscapes with high and diverse vegetation height yield greater benefits for foetal growth with compared to homogeneous landscapes [48]. For example, heterogenous landscapes which involve trees with diverse heights, as well as some gaps between trees, could be more beneficial for mothers than the grass fields or dense forests.

#### 4.3.5. Associations with Blue Space Measures

Four studies examined the associations between blue space measures and foetal growth [46,52,64,65]. A study [46] examined natural spaces including water bodies within 100 m of the residence of the mother as an exposure metric. The results indicated a statistically nonsignificant positive association between total natural spaces and increased BW; however, water bodies did not show any independent influence. Living within 1000 m proximity to coast was statistically significantly associated with a BW increase [52] and lower odds gestational diabetes [53]. The study also showed that living within 500 m of freshwater was associated with a 10.1 g (95% CI: 2.0, 18.2) increase in BW in the fully adjusted model, with a stronger association for women who were caucasian and lived in the wealthiest neighbourhoods. Other studies [64,65] did not find any statistically significant association between distance to water bodies and BW. 

Two studies [52,64] on the association between proximity to a freshwater body or coast and odds of PTB demonstrated no statistically significant association, except for those who live in the wealthiest neighbourhoods [52].

#### 4.3.6. Maternal Outcomes

There were relatively fewer publications on maternal outcomes. A study [51] reported that mothers who lived in greener areas were less likely (18%–23%) to report depressive symptoms, compared to those who lived in less green areas. Another study [37] did not observe association between proportion of green space and depressive symptoms, however, the trend was statistically significant for the proportion of green space within a 100 m buffer. A small but positive association was also observed within 300 m and 500 m buffers, when model adjusted for ethnicity. The study also found that women living within 300 m of a major green space were 13% less likely to report depressive symptoms than those living further than 300 m from major green space in adjusted models. There were no association between greenness and preeclampsia [45,70]. Similarly, only one study examined whether greenness within 500 m around the mother’s residence had a protective effect on maternal hypertension, and did not report any statistically significant association [69]. Also, there was no statistically significant relationship identified between green space and gestational diabetes in adjusted and unadjusted models [45]. Living within 500 m of a recreational place was statistically significantly associated with lower odds of gestational diabetes [53]. 

#### 4.3.7. Association of NDVI and Infant-Related Outcomes

An interquartile range increase in average NDVI within a 500 m buffer was statistically associated with an increase in 1.7 mm (0.5 mm, 2.9 mm) head circumference in unadjusted and adjusted models, while another study did not observe significant association [71]. The findings were robust for other buffer sizes (100 m and 250 m). In stratified analyses, stronger associations were observed among lower education groups. No statistically significant observation was found for an association between residential greenness and 5 min Apgar score [66]. An ecological study [27] found a small independent effect of greenness on decreased neonatal mortality.

### 4.4. Non-Linearities and Thresholds

A few studies observed non-linear associations between residential greenness and some birth outcomes, indicating inflection points in the association [51,60,61,67,68]. Non-linearity refers to a relationship between two variables, in which the alteration of the one variable is not directly proportional to the alteration of the other. The remaining studies did not consider non-linearities between greenness exposure and pregnancy outcomes. A study from the US [67] found a non-linear association between NDVI and BW, and a 0.1 NDVI increase between the 1st and 2nd quantiles was associated with a 6.69 g increase in BW, whereas NDVI increases between 2nd and 3rd quantiles resulted in a BW increase of 2.06 g. Mothers who lived in the greenest quintile (NDVI 0.60) and the 3rd and 4th quintile were 18% and 23% less likely to report depressive symptoms, respectively, when compared to those who live in the least quintile (NDVI 0.28) [51]. Another study [68] found that residing in the Q2 (0.37–0.45), Q3 (0.45–0.52) and Q4 (>0.52) NDVI quantile was associated with statistically significant increase of 2.7, 2.5 and 1.9 g BW, respectively. Relatedly, a study [61] also observed non-linearity between greenness and birth outcomes, and presented results Q4 compare to Q1, however, did not report inflection points. All studies suggest that moderate levels of greenness (NDVI) provided the maximum benefits on BW and depression. As in the case of green space, the quantity of blue space that is needed to promote healthier pregnancy outcomes remains unclear, because the studies did not report dose-response relationships.

Similarly, studies investigated residential tree effect on birth outcomes did not (or were unable to) consider associations with specific tree species [64]. Another study showed that particular type of natural area (accessible natural space, parks, woodlands, open water) did not have an individual effect on BW, while total natural space had a statistically significant protective effect on BW [46]. A recent study [39] observed the strongest association between neighbourhood greenness and birth outcomes when variance in greenness is at the highest level. 

### 4.5. Pathways and Effect Modifiers

Some pathways (reducing environmental pollution, lowering stress, providing settings for social and physical recreation) between green space and pregnancy outcomes were examined in a few studies [39,51,60]. Two studies found that physical activity partially mediated the influence of green space on depressive symptoms [51] and BW [39], while another study did not observe any mediation effect by hypothesized pathways [60]. Two studies have demonstrated that air pollution mediated the association between green space and maternal fasting glucose levels [41] and BW [43].

Some studies found effect modification by education level, SES, race and population density. Some studies [46,47,50,51,52,56,57,64,74] showed that the association between greenness level and birth outcomes was stronger for mothers with low and intermediate education, whereas other studies observed stronger association for mothers with higher education [61,67].

Some studies [39,49,57,64,74] identified a stronger association for women from the deprived population, while two [47,56] did not find any modification effect by SES. Furthermore, some studies [54,60,67] indicated that the high income groups have greater benefits from living in greener areas. 

Although two studies [47,56] found null results when investigating the effect modification by ethnicity, three reported differences by maternal race/ethnicity [49,61,68]. A study of Hispanic mothers [68] found a positive association between greenness and birth outcomes, but also reported a negative association among Black mothers. A similar result was reported by another study [49], where there was a protective association between greenness and BW among White British mothers, whereas no association was observed for Pakistani British mothers. 

Studies showed that benefits of greenness on TLBW [67], BW [57,60,61] and PTB [52] were stronger among women living in densely populated areas. Only one study [63] revealed that roadside trees can buffer the adverse effects of traffic related air pollution on the odds of TLBW. Other studies did not observe a modification effect by air pollution [10,39,47,57,61,67,70,75] or noise pollution [51].

The strongest association between living proximity to a freshwater body and BW was observed among mothers who were caucasian and from the wealthiest neighbourhood [52]. Similarly, the study also found that living within 500 m of the freshwater body was statistically significantly associated with a reduction in odds of PTB risk among mothers live in the high SES neighbourhoods.

## 5. Discussion

This systematic review and meta analysis reported evidence of higher BW and marginally lower odds of SGA with a higher residential green space. Limited evidence was found for the association between blue space and birth outcomes. No convincing evidence of association was observed between green space and odds of LBW and PTB. There are several possible explanations for these results; (1) few studies examined LBW and PTB that could reduce model power; (2) binary outcomes (e.g., LBW, PTB) provide less model power in comparison to continuous outcomes (e.g., BW); (3) the sample size of the studies might not be large enough to get a significant effect on the binary outcomes. However, these interpretations may not be applicable to SGA. The review has also shown consistent results on the beneficial health effects of surrounding green space and tree canopy on foetal growth; however, findings for gestation length and maternal outcomes were not conclusive. In addition, higher levels of greenness were associated with increased head circumference [76], reduced odds of neonatal mortality [27] and decreased likelihood of depressive symptoms in pregnant women [51]. Furthermore, a study [52] posited that babies of mothers who lived closer to blue space was heavier at birth. 

A few studies observed a positive nonlinear association between NDVI and BW [39,61,67,68] and the mental health of the mother [51], however, other studies did not consider non-linearity. Some studies [39,61,67,68] found that mothers who lived in an environment with a moderate quantity of green space had the lowest risk of having depressive symptoms during pregnancy, as well as giving birth to the heaviest babies, while those who lived in the greenest areas were associated with a positive but smaller improvement in these outcomes. These results are likely to be related to the following conditions that are likely context-dependent; (1) a larger quantity of green space might be a marker of an unfavourable urban form such as urban sprawl. Urban sprawl is a development pattern that is usually characterized by low density development that is associated with several adverse health outcomes, such as obesity [77], plausibly by way of triggering sedentary behaviours due to a longer walking distance between daily destinations. Sprawl pattern was also found to be linking to reduced social interaction [78], potentially due to inadequate public spaces where people would meet and participate to a collective activity. (2) The quality of green space might be more important than quantity of green space for pregnant women. For example, a small amount of good quality green spaces might benefit mothers more than greater amount of low quality green spaces would do. However, due to the unavailability of information on quality, we are unable to draw a firm conclusion. 

It warrants noting that some studies in this systematic review could not be included in the meta analysis. Reasons of excluding some studies from meta analysis were; (1) the small number (<5) of studies being available for some outcomes (e.g., head circumference, Apgar score) and exposures (e.g., blue space); (2) some information (e.g., standard deviation of outcomes) was not reported; (3) green space was not the main exposure [63]. 

All the studies measured green space via objective tools, however, no study considered the subjective measures of green or blue space quality. Mostly, cartographical databases of land use, or remotely sensed land use data, are used to classify density or distance to green space that perceived green space as a homogeneous environment type, and may lead to substantial misclassification, as these measures do not provide information regarding type and quality. A novel finding [39] showed that the increase in variance in green space that is potentially a marker of mix-land use had a protective effect on birth outcomes. The literature has shown that people prefer heterogeneous landscapes [48] within the context of urban environments, especially those containing green and blue spaces [25]. However, vegetation heterogeneity was not associated with birth outcomes [48]. The majority of the studies controlled for mother and infant related covariates in the statistical models. However, few studies considered other neighbourhood characteristics and environmental stressors. Thus, it remains unclear how much of what type of green space supports healthier birth outcomes. Evidence on this and on what qualities of green and blue spaces promote health of mothers and newborn babies is needed. 

Despite there being a theoretical basis on the potential pathways between green and blue spaces and birth outcomes, studies of candidate mediators also remains scarce. Physical activity was partly linked to the beneficial impact of green space on depressive symptoms [51] and BW [39]. Additionally, two studies have demonstrated that air pollution partly mediated the association between maternal glucose levels [41] and BW [43]. In contrast, a study [60] has shown that hypothesized pathways did not explain how green space influenced birth outcomes. Future studies may consider explicit tests of potential mechanisms to advance the field. 

Evidence of effect modification by SES was evident, with more favorable levels of association with green and blue space among mothers in socioeconomically disadvantaged circumstances (e.g., [13]). These disproportionate returns on investment may result from green space taking on greater salience for mothers in disadvantaged circumstances, who tend to lack the opportunities, autonomy, control over resources and capacities for adaptation that mothers in more affluent circumstances are likely to have [79]. Similar results have been found in studies of mortality [80] and mental health [81].

Overall, the generalisability of these findings might be problematic, due to several reasons: (1) the low number of studies; (2) the majority of studies were from high-income western countries and; (3) a reliance upon studies of cross-sectional design. Furthermore, we identified a considerable publication bias that is likely to lower the internal validity.

## 6. Strengths, Limitations and Future Research Directions

This review provides a synthesis of the most recent findings on green space and birth outcomes and, for the first time, integrates blue space as a potentially consequential exposure. Methodologically, this is an important advance, as the prescence of blue space may not only offer opportunities for the restoration and mitigation of environmental harms (e.g., air pollution), but also potentially enhances associations between green space and pregnancy outcomes via increasing quality. The limited number of studies found on how blue space relates to birth outcomes and modifies the associations with green space is an important finding of this review, and a gap that future studies are needed to resolve. 

There is no standard definition of exposure to green and blue space that is consistently used among the studies. Therefore, the heterogeneity in assessing green and blue space exposure across the studies has been main challenge for our review. Secondly, a meta-analysis was not feasible for some exposures (e.g., blue space) and outcomes (e.g., maternal depression), since few studies were available. Also, we conducted a meta-analysis for the effect estimates of the models that adjusted for the maximum number of covariates, however, there were differences in which covariates the studies adjusted for. 

This study has shown that the presence of green space in mother’s living environment has a positive influence on birth outcomes. However, it is still unclear what is the optimum amount, quality and type of green space which helps to promote positive birth outcomes. Therefore, investigations on identifying the optimum amount of green space, quality and also type are warranted. The identification of optimum amounts of green space could be possible by performing statistical methods, which can identify potential non-linear relationships, such as general additive models and using quantifable exposure measures, such as percent of green space and/or green space per capita. 

Green and blue space quality were also notable methodological gaps. Data on quality can be obtained by using objective and subjective quality measures, which could be obtained via public open space evaluation tools, such as the public open space desktop auditing tool (POSDAT), or conducting a survey to collect the user’s subjective perceptions. Information on the types of greenspaces (e.g., trees, urban parks) can be collected through using remote sensing technologies, which provide 3D metrics such as light detection and ranging technology (LIDAR). Addressing the aforementioned gaps could plausibly help guide urban planning strategies and public health policies in (re)designing cities to support improving public health in urban areas, such as by reducing the prevalance of adverse birth outcomes. 

Incorporating time-activity patterns or including places other than residence where the mother may spend time (e.g., work address) should also be considered. Potential pathways and effect modifiers (e.g., social contacts, population density, distance to the nearest major road) ought to be given full consideration to explore how green and blue space and pregnancy outcomes are influenced by other components of the natural and built environment. Finally, the current evidence is mostly from high-income countries. Studies to investigate associations in low-middle income countries will provide a clearer picture of what works for diverse populations with poor infrastructure, lower socio-economic levels and higher levels of air pollution.

## 7. Conclusions

In this review, we aimed to determine the threshold quantity of green and blue spaces to promote healthier birth outcomes. Although evidence continues to accumulate, non-linearities and threshold values remain under-researched. Additionally, we still do not know the influence of specific qualities of different type of green spaces on birth outcomes. Some evidence suggests a moderate level of green space could have stronger benefits compared to high or low levels. Meta-analysis showed that NDVI within an approximate 300 m buffer is positively associated with an increase in BW and lower odds of SGA. However, the potential pathways through which green and blue spaces influence birth outcomes remain complex and under-theorised. Future studies need to assess for nonlinear associations with effect modifiers and mediators of various types of green and blue space, as well as measures that describe different aspects of green and blue space quality, to advance the field of inquiry in a more consequential direction.

## Figures and Tables

**Table 1:** Meta-analysis results for the effect of Normalized Difference Vegetation Index (NDVI) increase on birth weight (standardized regression coefficients, quality effects model); LCI–Lower95%CI; UCI–Upper95%CI; β- standardized regression coefficient.

Study	β	LCI 95%	UCI 95%	Weight (%)
2019, Yin	0.0004	0.0012	−0.0003	3.79
2019, Dzhambov et al. (UIT)	0.0163	−0.0010	0.0340	0.65
2019, Dzhambov et al. (BBT)	0.0024	−0.0080	0.0130	0.66
2019, Eriksson et al.	0.0009	−0.0060	0.0080	0.68
2019, Nieuwenhuijsen et al.	0.0056	0.0041	0.0072	1.42
2018, Agay Shay et al.	0.0011	0.0006	0.006	10.30
2018, Cusack et al.	0.0028	−0.0026	0.0082	0.78
2018, Fog C. et al.	0.0005	0.0003	0.0007	39.45
2018, Glazer et al.	0.0021	−0.0001	0.0042	1.10
2017, Cusack et al. (Portland)	−0.0008	−0.0020	0.0004	1.98
2017, Cusack et al. (Austin)	0.0004	−0.0010	0.0017	1.73
2017, Abelt et al.	0.0019	0	0.0038	1.10
2015, Grazuleviciene et al.	0.0014	−0.0008	0.0037	0.92
2014, Agay-Shay et al.	0.0018	0.0012	0.0023	6.61
2016, Cusack et al.	0.0004	0	0.0008	13.42
2016, Casey et al.	0.0043	−0.0001	0.0088	0.84
2014, Hystad et al.	0.0035	0.0028	0.0042	5.10
2014b, Dadvand et al.	0.0037	0.0004	0.0070	0.88
2013, Markevych et al.	0.0039	0.0002	0.0076	0.85
2013, Laurent et al.	0.0001	−0.0004	0.0007	6.17
2012b, Dadvand et al.	0.0087	0.0004	0.017	0.67
2012a, Dadvand et al.	−0.0011	−0.0040	0.0015	0.88
Pooled	0.001	0.0002	0.0020	100
Statistics				
I-squared	86.4852	80.8324	90.4710	
Cochran’s Q	155.3858			
Chi2, p	0			
Q-Index	15.6628			

UIT: A Survey conducted in the Lower Inn Valley. Austria in 1998; BBT: A Survey conducted in the Wipp Valley, Austria and Italy.

**Table 2:** Meta-Analysis Results for the effect of NDVI increase on small for gestational age (odds ratios, quality effects model); LCI—Lower 95%CI; UCI—Upper 95%CI; OR—odds ratio.

Study	OR	LCI 95%	UCI 95%	Weight (%)
2019, Dzhambov et al. (UIT)	0.41	0.21	0.81	1.40
2019, Dzhambov et al. (BBT)	0.78	0.50	1.20	1.41
2019, Donovan et al.	0.94	0.90	1.00	2.36
2019, Eriksson et al.	0.31	0.10	1.00	1.40
2018, Agay-Shay et al.	0.95	0.87	1.03	2.44
2018, Fong C. et al.	0.98	0.97	0.99	30.00
2018, Glazer et al.	0.99	0.90	1.10	1.85
2017, Abelt et al.	1.17	0.89	1.54	1.43
2016, Cusack et al.	0.99	0.97	1.00	17.91
2016, Casey et al.	0.73	0.58	0.97	1.66
2015, Grazuleviciene et al.	0.93	0.81	1.08	1.46
2014, Hystad et al.	0.95	0.91	0.99	3.71
2012a, Dadvand et al.	0.99	0.98	1.00	32.97
Pooled	0.95	0.92	0.97	100.00
Statistics				
I-squared	56.99	20.17	76.82	
Cochran’s Q	27.90			
Chi2, p	0.01			
Q-Index	20.10			

**Table 3:** Meta-Analysis Results for the effect of NDVI increase on low birth weight (odds ratios, quality effects model); LCI—Lower95%CI; UCI—Upper95%CI; OR—odds ratio.

Study	OR	LCI 95%	UCI 95%	Weight (%)
2019, Yin	0.99	0.95	1.04	3.55
2019, Laurent et al.	0.96	0.95	0.98	27.64
2019, Dzhambov et al. (UIT)	0.43	0.23	0.81	0.94
2019, Dzhambov et al.(BBT)	0.75	0.47	1.20	0.95
2019, Nieuwenhuijsen et al.	0.80	0.70	0.90	1.41
2018, Agay Shay et al.	0.91	0.82	1.01	1.58
2018, Fong et al.	0.98	0.97	0.99	58.33
2017, Abelt, K., & McLafferty	1.08	0.63	1.83	0.95
2015, Grazuleviciene Et al.	0.94	0.69	1.29	0.94
2014, Agay-Shay Et al.	0.85	0.81	0.89	3.72
Pooled	0.96	0.91	1.01	100.00
Statistics				
I-squared	83.30	70.74	90.46	
Cochran’s Q	53.88			
Chi2, p	0.00			
Q-Index	9.90			

**Table 4:** Meta-Analysis Results for the effect of NDVI increase on preterm birth (odds ratios, quality effects model); LCI—Lower95%CI; UCI—Upper95%CI; OR—odds ratio.

Study	ES	LCI 95%	HCI 95%	Weight (%)
2019, Dzhambov et al. (UIT)	0.78	0.53	1.13	0.83
2019, Dzhambov et al. (BBT)	1.31	0.91	1.88	0.83
2018, Agay-Shay et al.	0.95	0.87	1.03	2.52
2018, Glazer et al.	0.99	0.90	1.09	1.77
2017, Abelt et al.	0.84	0.64	1.11	0.87
2016, Cusack et al.	1.01	0.99	1.02	47.98
2016, Casey et al.	0.78	0.61	0.99	1.04
2015, Grazuleviciene et al.	1.06	0.89	1.25	0.99
2014, Agay-Shay	1.01	0.97	1.05	6.19
2014, Hystad et al.	0.95	0.91	0.99	6.41
2013, Laurent et al.	0.98	0.97	1.00	30.56
Pooled	0.99	0.97	1.02	100.00
Statistics				
I-squared	53.58	8.17	76.53	
Cochran’s Q	21.54			
Chi2, p	0.02			
Q-Index	9.69			

## Supplementary Materials

The following are available online at https://www.mdpi.com/1660-4601/17/8/2949/s1, Figure S1: Assumption on potential pathways between green and blue space and pregnancy Outcome, Figure S2: Selection process of included articles, Figure S3: Funnel plot on the studies evaluating the effect of residential greenness (NDVI) on birthweight, Figure S4: Doi plot on the studies evaluating the effect of residential greenness (NDVI) on birthweight, Figure S5: Funnel plot on the effect of residential greenness (NDVI) on low birth weight, Figure S6: Doi plot on the studies evaluating the effect of residential greenness (NDVI) on low birth weight, Figure S7: Funnel plot on the effect of residential greenness (NDVI) on small for gestational age, Figure S8: Doi plot on the effect of residential greenness (NDVI) on small for gestational age, Figure S9: Funnel plot on the effect of residential greenness (NDVI) on preterm birth, Figure S10: Doi plot on the effect of residential greenness (NDVI) on preterm birth; Table S1:Digital Search Strategy, Table S2: Quality Assessment, Table S3: Characteristics of studies, Table S4: Exposure and outcome variables used in articles, Table S5: Extracted data and effect size computations for meta-analysis on birth weight, Table S6: Effect size computations for meta-analysis on birth weight based on 10% increase in green space proxies, Table S7: Meta-analysis result for effect of 10% increase in green space proxies, Table S8: Effect size computations for meta-analysis on birth weight based on 0.1 unit increase in NDVI, Table S9: Meta-Analysis result for the effect of 0.1 unit NDVI increase on birthweight, Table S10: Extracted data and effect size computations for meta-analysis on low birth weight, Table S11: Effect size computations for meta-analysis on low birth weight based on 10% increase in green space proxies, Table S12: Meta-analysis result for the effect of 10% increase in green space proxies, Table S13: Effect size computations for meta-analysis on low birth weight based on 0.1 unit increase in NDVI, Table S14: Meta-analysis result for the effect of 0.1 unit increase in NDVI on low birth weight, Table S15: Extracted data and effect size computations for meta-analysis on small for gestational age, Table S16: Effect size computations for meta-analysis on small for gestational age based on 10% increase in green space proxies, Table S17: Meta-analysis results for the effect of 10% increase in green space proxies, Table S18: Effect size computations for meta-analysis on small for gestational age based on 0.1 unit increase in NDVI, Table S19: Meta-analysis result for the effect of 0.1 unit increase in NDVI on small for gestational age, Table S20: Extracted data and effect size computations for meta-analysis on preterm birth LCI- lower 95% confidence interval, Table S21: Effect size computations for meta-analysis on preterm birth based on 10% increase in green space proxies, Table S22: Meta-analysis result for the effect of 10% increase in residential greenness, Table S23: Effect size computations for meta-analysis on preterm birth based on 0.1 unit increase in NDVI, Table S24: Meta-analysis results of the effect of 0.1 unit increase in NDVI on preterm birth, Table S25: Meta-Analysis Results for the effect of NDVI increase on term birth weight, Table S26: Meta-Analysis Results for the effect of NDVI increase on birth weight that are adjusted for gestational age, Table S27: Meta-Analysis Results for the effect of NDVI increase on term low birth weight, Table S28: Meta-Analysis Results for the effect of NDVI increase on low birth weight that are adjusted for gestational age
